# Seek COVER: using a disease proxy to rapidly develop and validate a personalized risk calculator for COVID-19 outcomes in an international network

**DOI:** 10.1186/s12874-022-01505-z

**Published:** 2022-01-30

**Authors:** Ross D. Williams, Aniek F. Markus, Cynthia Yang, Talita Duarte-Salles, Scott L. DuVall, Thomas Falconer, Jitendra Jonnagaddala, Chungsoo Kim, Yeunsook Rho, Andrew E. Williams, Amanda Alberga Machado, Min Ho An, María Aragón, Carlos Areia, Edward Burn, Young Hwa Choi, Iannis Drakos, Maria Tereza Fernandes Abrahão, Sergio Fernández-Bertolín, George Hripcsak, Benjamin Skov Kaas-Hansen, Prasanna L. Kandukuri, Jan A. Kors, Kristin Kostka, Siaw-Teng Liaw, Kristine E. Lynch, Gerardo Machnicki, Michael E. Matheny, Daniel Morales, Fredrik Nyberg, Rae Woong Park, Albert Prats-Uribe, Nicole Pratt, Gowtham Rao, Christian G. Reich, Marcela Rivera, Tom Seinen, Azza Shoaibi, Matthew E. Spotnitz, Ewout W. Steyerberg, Marc A. Suchard, Seng Chan You, Lin Zhang, Lili Zhou, Patrick B. Ryan, Daniel Prieto-Alhambra, Jenna M. Reps, Peter R. Rijnbeek

**Affiliations:** 1 Department of Medical Informatics, Erasmus University Medical Center, Doctor Molewaterplein, 403015 GD Rotterdam, The Netherlands; 2 Fundacio Institut Universitari per a la recerca a l’Atencio Primaria de Salut Jordi Gol i Gurina (IDIAPJGol), Barcelona, Spain; 3 Department of Veterans Affairs, University of Utah, Salt Lake City, UT USA; 4 Department of Biomedical Informatics, Columbia University, New York, NY USA; 5 School of Public Health and Community Medicine, UNSW, Sydney, Australia; 6 Department of Biomedical Sciences, Ajou University Graduate School of Medicine, Suwon, Republic of Korea; 7 Department of Big Data Strategy, National Health Insurance Service, Wonju, Republic of Korea; 8 Tufts University School of Medicine, Institute for Clinical Research and Health Policy Studies, Boston, MA 02111 USA; 9 Independent Epidemiologist, OHDSI, Rotterdam, The Netherlands; 10 So Ahn Public Health Center, Wando County Health Center and Hospital, Wando, Republic of Korea; 11 Nuffield Department of Clinical Neurosciences, University of Oxford, Oxford, UK; 12 Centre for Statistics in Medicine, NDORMS, University of Oxford, Oxford, UK; 13 Department of Infectious Diseases, Ajou University School of Medicine, Suwon, Republic of Korea; 14 Center for Surgical Science, Koege, Denmark; 15 Faculty of Medicine, University of Sao Paulo, Sao Paulo, Brazil; 16 Clinical Pharmacology Unit, Zealand University Hospital, Roskilde, Denmark; 17 NNF Centre for Protein Research, University of Copenhagen, Copenhagen, Denmark; 18 Abbvie, Chicago, USA; 19 Real World Solutions, IQVIA, Cambridge, MA USA; 20 Janssen Latin America, Buenos Aires, Argentina; 21 Department of Veterans Affairs, Washington D. C, USA; 22 Vanderbilt University, Nashville, USA; 23 Division of Population Health and Genomics, University of Dundee, Dundee, UK; 24 School of Public Health and Community Medicine, Institute of Medicine, Sahlgrenska Academy, University of Gothenburg, Gothenburg, Sweden; 25 Department of Biomedical Informatics, Ajou University School of Medicine, Suwon, Republic of Korea; 26 Quality Use of Medicines and Pharmacy Research Centre, University of South Australia, Adelaide, Australia; 27 Janssen Research & Development, Titusville, NJ USA; 28 Bayer Pharmaceuticals, Bayer Hispania, S.L., Barcelona, Spain; 29 Department of Public Health, Erasmus University Medical Center, Rotterdam, The Netherlands; 30 Department of Biomedical Data Sciences, Leiden University Medical Center, Leiden, The Netherlands; 31 Department of Biostatistics, UCLA Fielding School of Public Health, University of California, Los Angeles, CA USA; 32 School of Public Health, Peking Union Medical College, Beijing, China; 33 Melbourne School of Public Health, The University of Melbourne, Melbourne, Victoria Australia

**Keywords:** Patient-level prediction modelling, COVID-19, Risk score

## Abstract

**Background:**

We investigated whether we could use influenza data to develop prediction models for COVID-19 to increase the speed at which prediction models can reliably be developed and validated early in a pandemic. We developed COVID-19 Estimated Risk (COVER) scores that quantify a patient’s risk of hospital admission with pneumonia (COVER-H), hospitalization with pneumonia requiring intensive services or death (COVER-I), or fatality (COVER-F) in the 30-days following COVID-19 diagnosis using historical data from patients with influenza or flu-like symptoms and tested this in COVID-19 patients.

**Methods:**

We analyzed a federated network of electronic medical records and administrative claims data from 14 data sources and 6 countries containing data collected on or before 4/27/2020. We used a 2-step process to develop 3 scores using historical data from patients with influenza or flu-like symptoms any time prior to 2020. The first step was to create a data-driven model using LASSO regularized logistic regression, the covariates of which were used to develop aggregate covariates for the second step where the COVER scores were developed using a smaller set of features. These 3 COVER scores were then externally validated on patients with 1) influenza or flu-like symptoms and 2) confirmed or suspected COVID-19 diagnosis across 5 databases from South Korea, Spain, and the United States. Outcomes included i) hospitalization with pneumonia, ii) hospitalization with pneumonia requiring intensive services or death, and iii) death in the 30 days after index date.

**Results:**

Overall, 44,507 COVID-19 patients were included for model validation. We identified 7 predictors (history of cancer, chronic obstructive pulmonary disease, diabetes, heart disease, hypertension, hyperlipidemia, kidney disease) which combined with age and sex discriminated which patients would experience any of our three outcomes. The models achieved good performance in influenza and COVID-19 cohorts. For COVID-19 the AUC ranges were, COVER-H: 0.69–0.81, COVER-I: 0.73–0.91, and COVER-F: 0.72–0.90. Calibration varied across the validations with some of the COVID-19 validations being less well calibrated than the influenza validations.

**Conclusions:**

This research demonstrated the utility of using a proxy disease to develop a prediction model. The 3 COVER models with 9-predictors that were developed using influenza data perform well for COVID-19 patients for predicting hospitalization, intensive services, and fatality. The scores showed good discriminatory performance which transferred well to the COVID-19 population. There was some miscalibration in the COVID-19 validations, which is potentially due to the difference in symptom severity between the two diseases. A possible solution for this is to recalibrate the models in each location before use.

**Supplementary Information:**

The online version contains supplementary material available at 10.1186/s12874-022-01505-z.

## Background

In early 2020 the growing number of infections due to the coronavirus disease 2019 (COVID-19) resulted in unprecedented pressure on healthcare systems worldwide and caused many casualties at a global scale. Although the majority of people had uncomplicated or mild illness (81%), some developed severe disease leading to hospitalization and oxygen support (15%) or fatality (4%) [1, 2]. This presented a challenge both in finding effective treatments as well as in identifying which patients were at high risk and as such would benefit from protective measures. The most common diagnosis in severe COVID-19 patients was pneumonia, other known complications included acute respiratory distress syndrome (ARDS), sepsis, or acute kidney injury (AKI) [1].

The WHO Risk Communication Guidance distinguished two categories of patients at high risk of severe disease: those older than 60 years and those with “underlying medical conditions”, which is non-specific [3]. Using general criteria to assess the risk of poor outcomes is a crude risk discrimination mechanism as entire patient groupings are treated homogeneously ignoring individual differences. Prediction models can quantify a patient’s individual risk and data-driven methods could help to identify risk factors that have been previously overlooked. However, a systematic review evaluating all available prediction models for COVID-19 [4] concluded that despite the large number of prediction models being developed for COVID-19, none were considered ready for clinical practice. These COVID-19 prediction models were criticized for i) being developed using small data samples, ii) lacking external validation, and iii) being poorly reported.

In this article, we describe a process of using a proxy disease to develop a prediction model for another disease. This can be used in situations where there is a data scarcity for the disease of interest. In this process a model is developed using big data from a proxy disease and then assessed in the target disease. This preserves all the target disease data for validation to provide a more robust and reliable assessment of model performance in the intended setting. This increases the evidence of the performance of a model in the target disease compared to if the same data had been used for development. We describe a use-case for this process using influenza data to develop a model in the early stages of the COVID-19 pandemic. It has been well documented that influenza and COVID-19 have significant differences [5, 6]. However, we aim to show that influenza data can be used to develop a well performing model that could have been transported and used in early COVID-19 cases. The extensive external validation of the influenza developed model in early COVID-19 cases will robustly demonstrate the performance in COVID-19 patients and show areas that need adjustment and the model’s limitations. The lessons learned from this study could be used to inform the development of early prediction models in future pandemics.

## Methods

We performed a retrospective cohort study to develop COVID-19 prediction models for severe and critical illness. This study is reported according to the Transparent Reporting of a multivariate prediction model for Individual Prediction or Diagnosis (TRIPOD) guidelines [7].

At the start of the pandemic, there was very limited data available to develop prediction models due to the novel nature of the disease. To overcome the shortcoming of small data, we investigated whether we could use a proxy disease to develop a prediction model. This allowed us to utilise all available COVID-19 data for model validation. We developed models using historical data from patients with influenza or flu-like symptoms to assess a patient’s individual risk of developing severe or critical illness following infection using readily available information (i.e. socio-demographics and medical history). The developed models were validated against COVID-19 patients to test whether the performance transferred between the two settings.

We developed COVID-19 Estimated Risk (COVER) scores to quantify a patient’s risk of hospital admission with pneumonia (COVER-H), hospitalization with pneumonia requiring intensive services or death (COVER-I), or fatality (COVER-F) due to COVID-19 using the Observational Health Data Sciences and Informatics (OHDSI) Patient-Level Prediction framework [8]. The research collaboration known as OHDSI has developed standards and tools that allow patient-level prediction models to be rapidly developed and externally validated following accepted best practices [9]. This allows us to overcome two shortcomings of previous COVID-19 prediction papers by reporting according to open science standards and implementing widespread external validation.

### Source of data

This study used observational healthcare databases from six different countries. All datasets used in this paper were mapped into the Observational Medical Outcomes Partnership Common Data Model (OMOP-CDM) [10]. The OMOP-CDM was developed for researchers to have diverse datasets in a consistent structure and vocabulary. This enables analysis code and software to be shared among researchers, which facilitates replication and external validation of the prediction models.

The OMOP-CDM datasets used in this paper are listed in Table 1. All COVID-19 data was collected prior to 4/27/2020.

**Table 1:** Data sources formatted to the Observational Medical Outcomes Partnership Common Data Model (OMOP-CDM) used in this research (data type: claims, electronic health/medical records (EHR/EMR), general practitioner (GP))

Database	Database Acronym	Country	Data type	Contains COVID-19 data?	Time period
Columbia University Irving Medical Center Data Warehouse	CUIMC	United States	EMR	Yes	Influenza: 1990-2020 COVID-19: March-April 2020
Health Insurance and Review Assessment	HIRA	South Korea	Claims	Yes	COVID-19: 1^st^ January- 4^th^ April 2020
The Information System for Research in Primary Care	SIDIAP	Spain	GP and hospital admission EHRs linked	Yes	Influenza: 2006-2017 COVID-19: March 2020
Tufts Research Data Warehouse	TRDW	United States	EMR	Yes	Influenza: 2006-2020 COVID-19: March 2020
Department of Veterans Affairs	VA	United States	EMR	Yes	Influenza: 2009-2010, 2014-2019 COVID-19: 1^st^ March- 20^th^ April
Optum© De-Identified ClinFormatics® Data Mart Database^a^	ClinFormatics	United States	Claims	No	2000-2018
Ajou University School of Medicine Database	AUSOM	South Korea	EHR	No	1996 - 2018
Australian Electronic Practice based Research Network	AU-ePBRN	Australia	GP and hospital admission EHRs linked	No	2012-2019
IBM MarketScan® Commercial Database	CCAE	United States	Claims	No	2000-2018
Integrated Primary Care Information	IPCI	Netherlands	GP	Yes	2006-2020
Japan Medical Data Center	JMDC	Japan	Claims	No	2005-2018
IBM MarketScan® Multi-State Medicaid Database	MDCD	United States	Claims	No	2006-2017
IBM MarketScan® Medicare Supplemental Database	MDCR	United States	Claims	No	2000-2018
Optum^©^ de-identified Electronic Health Record Dataset	Optum EHR	United States	EHR	No	2006-2018

^a^Development database

### Participants

For model development, we identified patients aged 18 or older with a general practice (GP), emergency room (ER), or outpatient (OP) visit with influenza or flu-like symptoms (fever and either cough, shortness of breath, myalgia, malaise, or fatigue), at least 365 days of prior observation time, and no symptoms in the preceding 60 days. The initial healthcare provider interaction was used as index date, which is the point in time a patient enters the cohort.

For validation in COVID-19 we used a cohort of patients presenting at an initial healthcare provider interaction with a GP, ER, or OP visit with COVID-19 disease. COVID-19 disease was identified by a diagnosis code for COVID-19 or a positive test for the SARS-COV-2 virus that was recorded after 1/1/2020. We required patients to be aged 18 or over, have at least 365 days of observation time prior to the index date and no diagnosis of influenza, flu-like symptoms, or pneumonia in the preceding 60 days.

### Outcome

We investigated three outcomes: 1) hospitalization with pneumonia from index up to 30 days after index, 2) hospitalization with pneumonia that required intensive services (ventilation, intubation, tracheotomy, or extracorporeal membrane oxygenation) or death after hospitalization with pneumonia from index up to 30 days after index, and 3) death from index up to 30 days after index. Note that death is included in the second outcome to avoid incorrectly classifying patients who died without receiving intensive services as “low risk”.

The analysis code used to construct the participant cohorts and outcomes used for development and validation can be found in the R packages located at: https://github.com/ohdsi-studies/Covid19PredictionStudies

### Sensitivity analyses

We performed sensitivity analyses which involved using different versions of the COVID-19 cohort with varying sensitivities and specificities. At the beginning of the pandemic less testing capacity was available and as such we wanted to try broader definitions. Hence, we investigated three additional definitions where we included patients with symptoms, influenza, and visits any time prior to 2020. We then performed identical analysis with these changed cohorts.

### Predictors

We developed a data-driven model using age in groups (18–19, 20–25, 26–30, …, 95+), sex, and binary variables indicating the presence or absence of recorded conditions and drugs any time prior to the index date. Missing records are thus effectively imputed as zero, exceptions are age and sex, which are always recorded in the OMOP-CDM. In total, we derived 31,917 candidate predictors indicating the presence of unique conditions/drugs recorded prior to the index date (GP, ER, or OP visit) for each patient. When using a data-driven approach to model development, generally the resulting models contain many predictors. This may optimise performance, but can be a barrier to clinical implementation. The utility of models for COVID-19 requires that they can be widely implemented across worldwide healthcare settings. Therefore, in addition to a data-driven model, we investigated two models that include fewer candidate predictors.

The age/sex model used only age groups and sex as candidate predictors. The COVER scores used a reduced set of variables, which were obtained by the following process:

1. Multiple clinicians inspected the data-driven model to identify variables that had a high standardized mean difference between patients with and without the outcome calculated using the following equation

$$\left( standardisedMeanDifference=\frac{mean\ with\ outcome- mean\ with out\ outcome}{\sqrt{variance\ with\ outcome+ variance\ with out\ outcome}}\right).$$

There are often multiple predictors which are related and correlated selected by the model, for example a model might select a condition occurrence in different time periods predating the index date. This could be simplified to one predictor saying only “Patient had condition X in history”, instead of having multiple predictors specifying in which time period the condition occurred. Likewise, multiple codes that are probably related to a specific condition could be simplified in one predictor. We identified general categories from these such as ‘heart disease’ and ‘diabetes’.

2. Phenotype definitions for each category were created. This was performed to make the definitions clinically meaningful.
3. We trained a LASSO logistic regression model on the original data using age groups, sex and the newly created predictors indicating whether the patient had any of the category predictors.
4. The coefficients of this reduced variable model were then multiplied by 10 and rounded to the nearest integer. This was done to make the model simpler to calculate.
5. This gave us the simple score-based model.

### Sample size

**Fig. 1:**
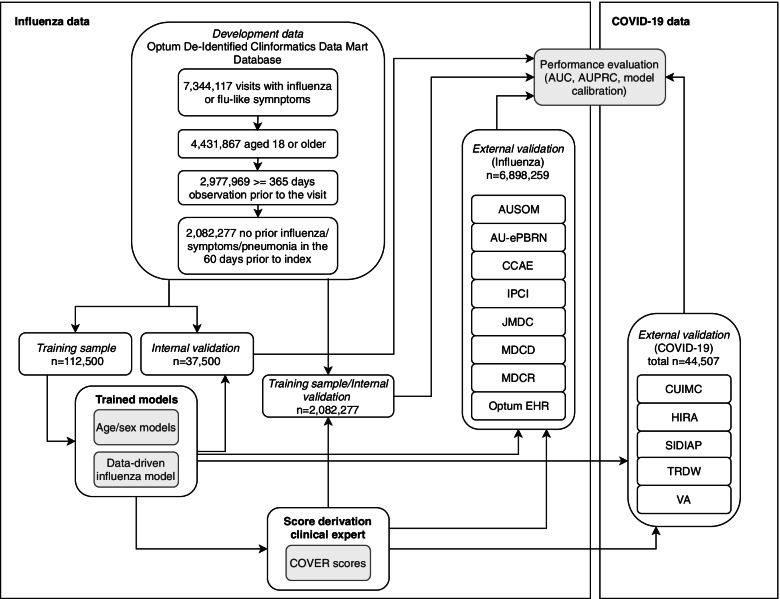
A Flow chart representing the path of data in the study. This details the splits used internally for model development, the steps taken for model parsimonisation and validation and external validation

The models were developed using the Optum© De-Identified ClinFormatics® Data Mart Database. We identified 7,344,117 valid visits with influenza or flu-like symptoms, of which 4,431,867 were for patients aged 18 or older, 2,977,969 of these had at least 365 days of prior observation time, and 2,082,277 of these had no influenza/symptoms/pneumonia in the 60 days prior to index. We selected a random sample of 150,000 patients from the total population, as research showed it is possible to efficiently develop models with near optimal performance, while reducing model complexity and computational requirements by using a sample of this size [11]. Riley et al. provide a calculator for minimum sample size, which for number of predictors = 20, event rate = 0.05 and R^2^ = 0.1 would require a minimum of 1698 patients [12]. This subset was used to develop the data-driven model. The full set of 2,082,077 patients was then used for the development and validation of the simple model. A small subset of this data was used to develop the data-driven model and so the presented internal performance could be optimistic. In theory this is a limitation, but it has no effect on the evidence of the external validation. Fig. 1 is a flow chart demonstrating the above exclusions and flow of data through the study.

### Missing data

Age and sex are required by the OMOP-CDM used by OHDSI and will never be missing. For each condition or drug we considered no records in the database to mean the patient does not have the condition or does not receive the drug. This could lead to misclassification of patients if a patient’s illness is not recorded in the database.

### Statistical analysis methods

Model development followed a previously validated and published framework for the creation and validation of patient-level prediction models [8]. We used a person ‘train-test split’ method to perform internal validation. In the development cohort, a random split sample (`training sample’) containing 75% of patients was used to develop the prediction models and the remaining 25% of patients (`test sample’) was used to internally validate the models. We trained models using LASSO regularized logistic regression, using a 3-fold cross validation technique in the train-set to learn the optimal regularization hyperparameter through an adaptive search [13]. We used R (version 3.6.3) and the OHDSI Patient-Level Prediction package (version 3.0.16) for all statistical analyses [8].

To evaluate the performance of the developed models, we calculate the overall discrimination of the model using the area under the receiver operating characteristic curve (AUC), the area under the precision recall-curve (AUPRC), and the model calibration. The AUC indicates the probability that for two randomly selected patients, the patient who gets the outcome will be assigned a higher risk. The AUPRC shows the trade-off between identifying all patients who get the outcome (recall) versus incorrectly identifying patients without outcome (precision) across different risk thresholds. The model calibration is presented in a plot to examine agreement between predicted and observed risks across deciles of predicted risk. Calibration assessment is then performed visually rather than using a statistic or numeric value as this provides a better impression of the direction and scale of miscalibration [14]. Summary statistics are reported from the test samples.

We performed external validation in databases containing COVID-19 data. To do this we assessed patients with confirmed COVID-19. In addition, we performed a classical external validation in which we applied the models to identical settings across diverse patient populations with influenza or flu-like symptoms prior to 2020. We examined the external validation using AUC, AUPRC and model calibration in the same way as internally. We provide confidence intervals when the number of events is below 1000. Once the number of events increases, confidence intervals become too narrow to provide a good estimate of error.

This study adheres to open science principles for publicly prespecifying and tracking changes to study objectives, protocol, and code as described in the Book of OHDSI [15]. For transparency, the R packages for the development and external validation of the models in any database mapped to the OMOP-CDM are available on GitHub at:

https://github.com/ohdsi-studies/Covid19PredictionStudies

## Results

### Online results

The complete results are available as an interactive app at: http://evidence.ohdsi.org/Covid19CoverPrediction

This application will continue to be updated as the models are validated, an archived version of the app that was released to accompany this article is available here: https://zenodo.org/record/4697417

### Participants

Table 2 describes the characteristics at baseline of the patients across the databases used for development and external validation. Out of the 150,000 patients sampled with influenza or flu-like symptoms in the development database (ClinFormatics), there were 6712 patients requiring hospitalization with pneumonia, 1828 patients requiring hospitalization and intensive services with pneumonia or death, and 748 patients died within 30 days. See Table 2 for the full outcome proportions across the databases included in this study. A total of 44,507 participants with COVID-19 disease were included for external validation.

**Table 2:** Population size, outcome proportion, and characteristics for the development database (influenza) and external validation databases for COVID-19 and influenza (N/A indicates this result is not available)

	Development	External validation: COVID-19					External validation: influenza							
	ClinFormatics	CUIMC	HIRA	SIDIAP	TRDW	VA	AUSOM	AU-ePBRN	CCAE	IPCI	JMDC	MDCD	MDCR	Optum EHR
Number of participants	2,082,277	2,731	1,985	37,950	395	1,446	3,105	2,791	3,146,801	29,132	1,276,478	536,806	248,989	1,654,157
Hospitalization with pneumonia (Outcome proportion %)	105,030 (5.04)	N/A	89 (4.48)	1,223 (1.11)	21 (5.32)	149 (10.30)	49 (1.58)	29 (1.04)	33,824 (1.07)	22 (0.08)	728 (0.06)	32,987 (6.15)	31,059 (12.47)	34,229 (2.07)
Hospitalization with pneumonia requiring intensive services or death (Outcome proportion %)	29,905 (1.44)	134 (4.91)	22 (1.11)	N/A	5 (1.27)	38 (2.63)	5 (0.16)	3 (0.11)	4,856 (0.02)	24 (0.08)	65 (0.01)	7,226 (1.35)	3,628 (1.46)	7,368 (0.45)
Death (Outcome proportion %)	11,407 (0.55)	335 (12.27)	43 (2.17)	406 (1.07)	1 (0.25)	43 (2.97)	5 (0.16)	4 (0.14)	965 (0.03)	24 (0.08)	75 (0.01)	2,603 (0.48)	1,354 (0.54)	3,513 (0.21)
Age (% above 65)	26.1	38.9	15.6	17.9	18.2	37.3	11.9	23.1	12.5	16.9	16.0	14.2	96.2	30.0
Sex (%, male)	44.4	47.2	43.5	43.4	49.6	81.4	41.7	44.5	42.7	43.7	56.8	29.2	45.9	40.1
Cancer (%)	12.6	17.1	9.8	6.3	11.6	17.0	7.7	8.2	6.2	3.7	2.5	8.9	35.2	10.6
COPD (%)	10.2	9.3	4.9	2.5	6.3	20.5	2.7	3.1	2.7	2.7	0.5	19.8	26.6	7.6
Diabetes (%)	20.5	30.9	23.1	8.0	19.7	35.2	3.8	13.0	11.4	6.7	8.3	27.4	36.1	15.3
Heart disease (%)	31.0	40.1	17.1	11.2	25.8	44.7	7.7	12.9	16.5	7.5	8.0	36.1	68.2	23.4
Hypertension (%)	44.2	51.6	26.3	14.8	38.5	63.0	13.9	27.0	29.1	12.4	11.4	49.8	80.4	36.1
Hyperlipidemia (%)	46.8	40.6	39.9	11.4	32.9	62.5	3.3	20.2	21.8	4.6	15.2	36.0	69.6	34.2
Kidney disease (%)	18.7	31.2	17.0	11.0	24.3	32.4	7.6	6.2	9.0	1.2	5.1	23.4	35.5	14.9

In the databases used for external validation, the patient numbers ranged from 395 (TRDW) to 3,146,743 (CCAE). The datasets had varied outcome proportions ranging from 0.06–12.47 for hospital admission, 0.01–4.91 for intensive services, and 0.01–12.27 for fatality. Characteristics at baseline differed substantially between databases as can be seen in Table 2, with MDCR (a database representing retirees) containing a relatively old population of patients and a high number of comorbidities, and IPCI (a database representing general practice) showing a relatively low condition occurrence.

### Model performance

**Fig. 2:**
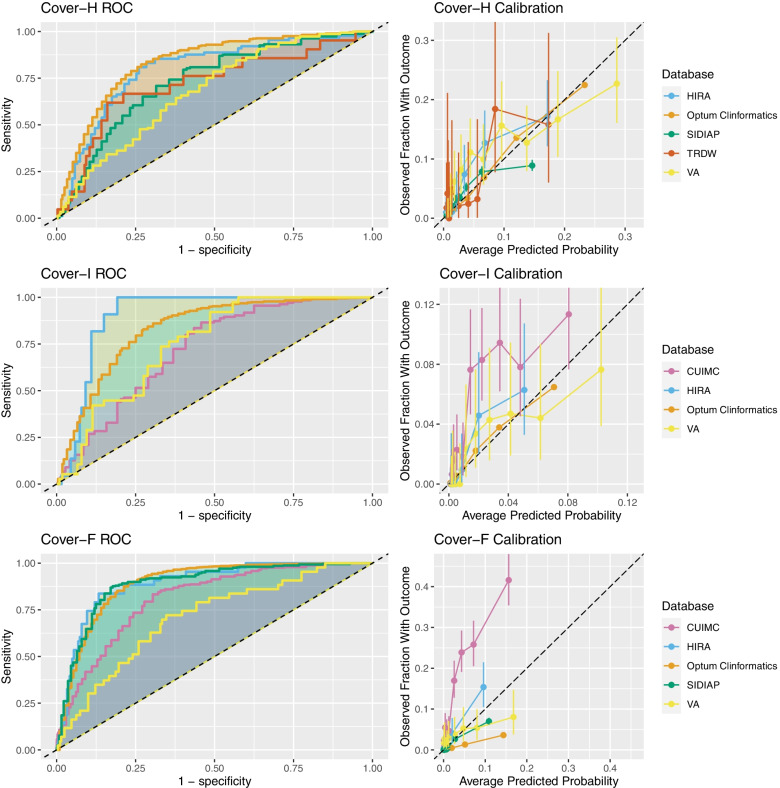
The ROC and Calibration plots for the validations (internal and external) of the 3 Cover scores

The internal validation performance for each model is presented in Table 3. The external validation of the COVER scores on the COVID-19 patients is shown in Table 4. Full validation results can be seen in Appendix 1B of the online supplement. Receiver operating characteristic and calibration plots are included in Fig. 2 and Appendix 1C of the online supplement.

**Table 3:** Results for internal validation in ClinFormatics

Outcome	Predictors	No. Variables	AUC	AUPRC
Hospitalization with pneumonia	Conditions/drugs + age/sex	521	0.852	0.224
	Age/sex	2	0.818	0.164
	COVER-H	9	0.840	0.120
Hospitalization with pneumonia requiring intensive services or death	Conditions/drugs + age/sex	349	0.860	0.070
	Age/sex	2	0.821	0.049
	COVER-I	9	0.839	0.059
Fatality	Conditions/drugs + age/sex	205	0.926	0.069
	Age/sex	2	0.909	0.037
	COVER-F	9	0.896	0.039

**Table 4:** Results of external validation of the COVER scores on COVID-19 patients with a GP, ER, or OP visit in 2020 (*Confidence interval is not reported as the number of outcomes is larger than 1000)

Outcome	Database	AUC (95% confidence interval)	AUPRC
Hospitalization with pneumonia (COVER-H)	HIRA	0.806 (0.762-0.851)	0.134
	SIDIAP	0.748*	0.072
	TRDW	0.731 (0.611-0.851)	0.132
	VA	0.689 (0.649-0.729)	0.179
Hospitalization with pneumonia requiring intensive services or death (COVER-I)	CUIMC	0.734 (0.699-0.769)	0.100
	HIRA	0.910 (0.889-0.931)	0.053
	VA	0.763 (0.708-0.818)	0.058
Fatality (COVER-F)	CUIMC	0.820 (0.796-0.840)	0.400
	HIRA	0.898 (0.857-0.940)	0.150
	SIDIAP	0.895 (0.881-0.910)	0.083
	VA	0.717 (0.642-0.791)	0.068

### Model specification

**Fig. 3:**
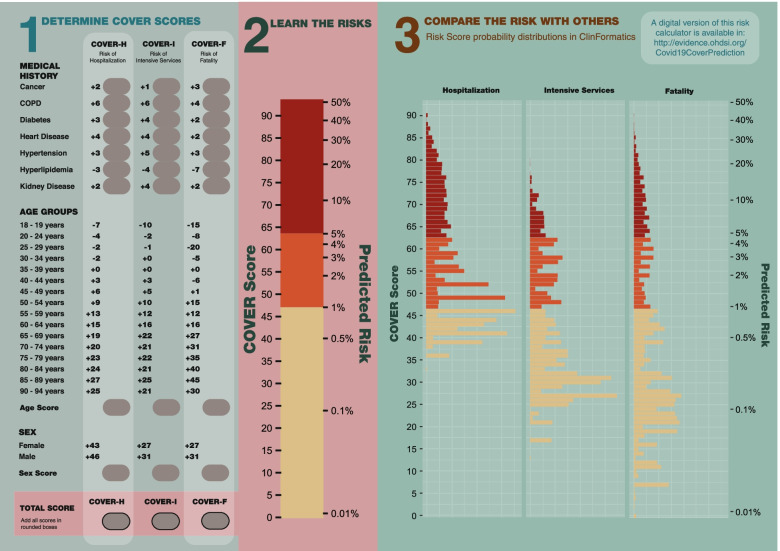
A graphic showing how to calculate the 3 Cover scores with a nomogram to convert the raw score into a percentage risk. There is also a distribution of scores found using internal validation to allow for comparison of a patients score to the wider population

The data-driven models for hospitalization, intensive services, and fatality contained 521, 349, and 205 predictors respectively. The COVER-H, COVER-I, and COVER-F scores are presented in Fig. 3. After data-driven selection, clinicians reviewed the resulting models and created the composite predictors. This produced the COVER scores which include 7 predictors, in addition to age groups and sex, that corresponded to the following conditions existing any time prior to the index date: cancer, chronic obstructive pulmonary disease, diabetes, heart disease, hypertension, hyperlipidemia, and kidney disease (chronic and acute). A description of the covariates can be found in Appendix 1A of the online supplement. The COVER scores are detailed in Fig. 3 and are accessible online under the calculator tab at: http://evidence.ohdsi.org:3838/Covid19CoverPrediction/

Figure 3 also provides a risk converter, which allows for easy conversion between the risk score and predicted risk of the outcomes. The scores can be converted to a probability by applying the logistic function: 1/(1 + exp.((risk score-93)/10)). Furthermore, we provide a plot of the probability distribution for each of the three models from patients in ClinFormatics to demonstrate the expected regions the probabilities fall into. To calculate the COVER scores using Fig. 3, a clinician first needs to identify which conditions the patient has. The points for the corresponding predictors are then added to arrive at the total score. For example, if a 63-year-old female patient has diabetes and heart disease, then her risk score for hospital admission (COVER-H) is 43 (female sex) + 4 (heart disease) + 3 (diabetes) + 15 (age) = 65. The risk scores for intensive services (COVER-I) and fatality (COVER-F) are 51 and 47, respectively. Using the risk converter in Fig. 3, a score of 65 corresponds to a risk of 6%. Scores of 51 and 47 correspond to 1.5 and 1%, respectively.

## Discussion

### Interpretation

We developed and externally validated models using large datasets of influenza patients to quantify a patient’s risk of developing severe or critical illness due to COVID-19. In the development data, the 9-predictor COVID-19 Estimated Risk (COVER) scores were a good trade-off between model complexity and performance, as the AUCs were generally close to the large data-driven models. In the development database the COVER scores achieved an AUC of 0.84 when predicting which patients will be hospitalized or require intensive services and an AUC of 0.90 when predicting which patients will die within 30 days. When validated on 1985 COVID-19 patients in South Korea the COVER-H score achieved an AUC of 0.81, COVER-I and COVER-F achieved an AUC of 0.90 and 0.91. When applied to 37,950 COVID-19 Spanish patients COVER-H had an AUC of 0.75 and performed better when predicting fatality (COVER-F: AUC 0.89). When applied to US patients, the COVER-I and COVER-F models achieved AUCs of 0.73 and 0.82 in CUIMC, VA performed similarly with AUCs of 0.76 and 0.72 respectively. The VA also achieved 0.69 for COVER-H. The results show reasonable performance with some inconsistency across a range of countries.

A visual assessment of calibration plots across validations showed reasonable calibration in HIRA, SIDIAP, and VA. There was a slight overestimation of risk amongst oldest and highest risk strata in SIDIAP, and to a lesser extent in HIRA. The calibration was poor in CUIMC, as risk was often underestimated. This may be due to CUIMC containing mostly hospitalized COVID-19 patients, so the CUIMC cohort are experiencing more severe COVID-19. The VA showed some miscalibration in the lowest and highest risk strata. The observed miscalibration is possibly due to the differing severities of the diseases used for model development and calibration. However, miscalibration could also be due to other differences in populations not caused by the use of a proxy disease. The variable calibration results suggest that the model’s performance should be assessed and the model should potentially be recalibrated before being implemented in a local context. A simple method to do this is by adjusting the baseline risk based upon the differences found between development and validation populations using an adjustment factor derived from the differences in case mix between development and validation settings [16, 17].

The age/sex models also show reasonable performance, and these predictors are among the main contributors to performance in the COVER scores. This suggests these models could also be suitable if access to medical history is difficult.

These results showed that training in large historical influenza data was an effective strategy to develop models for COVID-19 patients. We also performed sensitivity analyses using more sensitive COVID-19 definitions, for example including patients with symptoms, influenza, and visits any time prior to 2020. The results did not show much deviation from the specific definition (online supplement Appendix 1B). Our results show that quantifying a symptomatic patient’s risk based on a small selection of comorbidities as well as age/sex gives improved model performance.

### Limitations

First, it has become clear that there are differences in the underlying nature of the two diseases, particularly in respect to the severity of symptoms in COVID-19 patients compared with influenza patients. Therefore, it is possible another disease may have provided a better proxy than influenza.

Second, despite preserving all the target disease data for validation, we still had relatively low outcome numbers. In the CUIMC, HIRA, SIDIAP, and VA COVID-19 databases we either reached or approached the threshold for reliable external validation of 100 patients who experience the outcome of interest [18, 19], but the results of TRDW might not be reliable.

Furthermore, the data reported early during the COVID-19 pandemic was noisy and skewed. This might cause misclassification in the target and outcome cohorts. In order to counter this, we performed sensitivity analysis using cohorts that included broad and narrow COVID-19 definitions, the impact of this on the results was minimal. The use of a 30-day risk window has the limitation that if a patient experiences an outcome after the time window, this will be (incorrectly) recorded as a non-event. There is further potential misclassification of predictors, for example, if a disease is incorrectly recorded in a patient’s history. Moreover, the result of the phenotype generation process is not fully reproducible due to the use of clinician expertise, which is an unresolved problem in much epidemiological work. However, the phenotype development process is reproducible and the phenotypes generated are provided. The evidence in the paper shows the models to be robust and transportable.

We were unable to include some suspected disease predictors in the analysis as these are not readily available (e.g. lymphocyte count, lung imaging features) or inconsistently collected and reported across the various databases included in the study (e.g. BMI, ethnicity). However, due to the high load on healthcare systems and the contagious nature of the disease we believe it is useful to have a model that does not require a patient to be either in hospital or another setting to receive tests. A similar issue also meant we were not able to validate the COVER-H score in CUIMC (it mostly contains ER or hospitalized COVID-19 patients) and the COVER-I score in SIDIAP (due to a lack of information on intensive services in the database).

Finally, concerns exist over the clinical validity of claims data, however we were able to develop models using claims data that transported well into EHR data. There is the potential for some overlap of patients between claims and EHR databases, although this number is likely to be small.

### Implications

The results show we were able to develop models that use historical influenza patient’s socio-demographics and medical history to predict their risk of becoming severely or critically ill when infected with COVID-19. To our knowledge, this is the first study that has been able to extensively externally validate prediction models on COVID-19 patients at a global scale. The adequate performance of the COVER scores in COVID-19 patients (as quantified by consistent finding of AUC > 0.7 in new settings) show these scores could have been used to identify patients who should have been shielded from COVID-19 in the early stages of the pandemic.

## Conclusion

In this paper we developed and validated models that can predict which patients presenting with COVID-19 are at high risk of experiencing severe or critical illness. This research demonstrates that it is possible to develop a prediction model rapidly using historical data of a similar disease that, once re-calibrated with contemporary data and outcomes from the current outbreak, could be used to help inform strategic planning and healthcare decisions.

## Supplementary Information

**Additional file 1.**

**Additional file 2.**

## Data Availability

The datasets generated and/or analysed during the current study are not publicly available due to patient privacy and data protection concerns. Information on access to the databases is available from the corresponding author.
