# Supplementary material for: Seek COVER: using a disease proxy to rapidly develop and validate a personalized risk calculator for COVID-19 outcomes in an international network
Source: BMC Med Res Methodol. 2022 Jan 30;22:35. doi: 10.1186/s12874-022-01505-z (PMC8801189; doi:10.1186/s12874-022-01505-z)
Supplement: Supplementary file 1 — Additional file 1. [file 12874_2022_1505_MOESM1_ESM.docx]

# Appendix 1A: COVER covariate descriptions

In the Observational Medical Outcomes Partnership Common Data Model (OMOP-CDM) diagnoses are classified in hierarchies. This means that a certain diagnosis, or condition occurrence in the context of this document, has a number of descendants. Diabetes mellitus, for example, is a descendant of diabetes and has numerous, more detailed descendants such as type 1 diabetes mellitus, which can be illustrated like this:


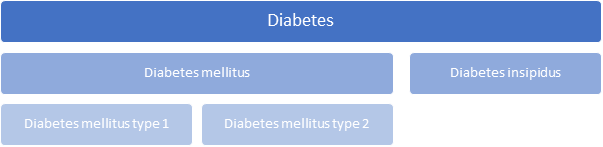


The actual hierarchical structure of SNOMED-CT, one of the classifications on which the OMOP-CDM rests, is more complex but the notion remains.

For most diseases, we cannot know the exact time of onset, and must use the point in time on which the condition was recorded (condition occurrence) as the proxy. Thus, within each of the following groups, a history of the condition in question is operationalised as at least one occurrence of the listed conditions at any time prior to index. The concept id's uniquely identify conditions and are made as hyperlinks taking the reader to the ATHENA interface (https://athena.ohdsi.org/) facilitating perusal of their hierarchical positions.

| **Condition occurrences** | **Examples** | **Concept id’s** |
| --- | --- | --- |
| History of cancer |  |  |
| Any malignant neoplastic disease or descendants | - Leukaemia - Large-cell carcinoma in lung | [443392](https://athena.ohdsi.org/search-terms/terms/443392/graph?levels=4&standardsOnly=true&zoomLevel=4) |
| Except squamous cell carcinoma |  | [4111921](https://athena.ohdsi.org/search-terms/terms/4111921/graph?levels=4&standardsOnly=true&zoomLevel=4) |
| Except basal cell carcinoma |  | [4112752](https://athena.ohdsi.org/search-terms/terms/4112752/graph?levels=4&standardsOnly=true&zoomLevel=4) |
| History of COPD |  |  |
| Chronic obstructive pulmonary disease or descendants | - Pulmonary emphysema - Acute exacerbation of COPD | [255573](https://athena.ohdsi.org/search-terms/terms/255573/graph?levels=4&standardsOnly=true&zoomLevel=4) |
| History of diabetes mellitus |  |  |
| Diabetes mellitus (including type 1 or 2) or descendants | - Type 1 diabetes mellitus - Type 2 diabetes mellitus | [201820](https://athena.ohdsi.org/search-terms/terms/201820/graph?levels=4&standardsOnly=true&zoomLevel=4) |
| Documented poor glycaemic control with/without complications | - Type 1 diabetes mellitus uncontrolled | [443238](https://athena.ohdsi.org/search-terms/terms/443238/graph?levels=4&standardsOnly=true&zoomLevel=4) |
| Complication due to diabetes mellitus |  | [442793](https://athena.ohdsi.org/search-terms/terms/442793/graph?levels=4&standardsOnly=true&zoomLevel=4) |
| History of heart disease |  |  |
| Heart disease or descendants | - Disease of coronary artery - Endocarditis | [321588](https://athena.ohdsi.org/search-terms/terms/321588/graph?levels=4&standardsOnly=true&zoomLevel=4) |
| History of hyperlipidaemia |  |  |
| Hyperlipidaemia or descendants | - Hypercholesterolemia - Hypertriglyceridemia | [432867](https://athena.ohdsi.org/search-terms/terms/432867/graph?levels=4&standardsOnly=true&zoomLevel=4) |
| History of hypertension |  |  |
| Hypertension or any descendants | - Essential hypertension - Benign hypertension | [316866](https://athena.ohdsi.org/search-terms/terms/316866/graph?levels=4&standardsOnly=true&zoomLevel=4) |
| History of kidney disease |  |  |
| Kidney disease or descendants. We consider both chronic and acute kidney disease together for a broader cohort definition. | - Acute nephropathy - Hypertensive renal disease | [198124](https://athena.ohdsi.org/search-terms/terms/198124/graph?levels=4&standardsOnly=true&zoomLevel=4) |

The phenotypes for each COVER predictor are available in Appendix 2.

# Appendix 1B: Full results

Due to differing data collection methods and policies not all databases could run all the models. For instance, IPCI, a GP database, does not contain information on intensive services, so we were able to make predictions of patients being hospitalised, but we were unable to make predictions of whether they would go on to receive intensive services. Some of the databases chose only to validate the models with limited covariates.

Supplementary Table 1 External validation of the models on the target population of patients with influenza or flu-like symptoms any time prior to 2020 (N/A indicates this result is not available)

| Outcome | Database | Conditions/drugs + age/sex | | Age/sex | | COVER | |
| --- | --- | --- | --- | --- | --- | --- | --- |
|  |  | AUC | AUPRC | AUC | AUPRC | AUC | AUPRC |
| Hospitalization with pneumonia | AUSOM | N/A | N/A | 0.760 | 0.056 | 0.768 | 0.061 |
|  | AU-ePBRN | N/A | N/A | N/A | N/A | 0.756 | 0.031 |
|  | CCAE | 0.769 | 0.073 | 0.690 | 0.024 | 0.728 | 0.040 |
|  | IPCI | 0.686 | 0.002 | 0.681 | 0.008 | 0.683 | 0.002 |
|  | JMDC | 0.686 | 0.007 | 0.645 | 0.002 | 0.660 | 0.003 |
|  | MDCD | 0.804 | 0.191 | 0.757 | 0.153 | 0.779 | 0.167 |
|  | MDCR | 0.681 | 0.225 | 0.633 | 0.195 | 0.660 | 0.207 |
|  | Optum EHR | 0.815 | 0.087 | 0.777 | 0.73 | 0.804 | 0.090 |
| Hospitalization with pneumonia requiring intensive services or death | AUSOM | 0.896 | 0.216 | 0.770 | 0.010 | 0.783 | 0.028 |
|  | AU-ePBRN | N/A | N/A | N/A | N/A | 0.923 | 0.007 |
|  | CCAE | 0.816 | 0.020 | 0.718 | 0.004 | 0.774 | 0.009 |
|  | IPCI | N/A | N/A | N/A | N/A | N/A | N/A |
|  | JMDC | 0.778 | 0.002 | 0.708 | 0.000 | 0.750 | 0.001 |
|  | MDCD | 0.802 | 0.048 | 0.741 | 0.030 | 0.773 | 0.037 |
|  | MDCR | 0.689 | 0.035 | 0.556 | 0.019 | 0.652 | 0.026 |
|  | Optum EHR | 0.832 | 0.024 | 0.770 | 0.014 | 0.814 | 0.020 |
| Fatality | AUSOM | 0.812 | 0.017 | 0.793 | 0.007 | 0.798 | 0.008 |
|  | AU-ePBRN | N/A | N/A | N/A | N/A | 0.893 | 0.007 |
|  | CCAE | 0.833 | 0.016 | 0.780 | 0.001 | 0.806 | 0.002 |
|  | IPCI | 0.866 | 0.020 | 0.856 | 0.008 | 0.859 | 0.008 |
|  | JMDC | 0.766 | 0.001 | 0.723 | 0.000 | 0.724 | 0.001 |
|  | MDCD | 0.842 | 0.027 | 0.823 | 0.022 | 0.829 | 0.022 |
|  | MDCR | 0.678 | 0.014 | 0.598 | 0.008 | 0.627 | 0.009 |
|  | Optum EHR | 0.889 | 0.024 | 0.867 | 0.018 | 0.872 | 0.016 |

Supplementary Table 2 COVID-19 validation of the COVER-H, COVER-I, and COVER-F scores (N/A indicates this result is not available)

| Outcome | Database | Patients with COVID-19, influenza or flu-like symptoms | | | Patients with COVID-19, influenza or flu-like symptoms in 2020 | | | Patients with COVID-19 or symptoms in 2020 | | | Patients with COVID-19 in 2020 | | |
| --- | --- | --- | --- | --- | --- | --- | --- | --- | --- | --- | --- | --- | --- |
|  |  | Number of participants (Outcome rate %) | AUC | AUPRC | Number of participants (Outcome rate %) | AUC | AUPRC | Number of participants (Outcome rate %) | AUC | AUPRC | Number of participants (Outcome rate %) | AUC | AUPRC |
| Hospitalization with pneumonia (COVER-H) | CUIMC | N/A | N/A | N/A | N/A | N/A | N/A | N/A | N/A | N/A | N/A | N/A | N/A |
|  | HIRA | 58,072  (4.61) | 0.767 | 0.132 | 48,057  (5.25) | 0.762 | 0.143 | 47,594  (5.18) | 0.763 | 0.142 | 1,985  (4.48) | 0.806 | 0.134 |
|  | SIDIAP | 415,119 (0.12) | 0.697 | 0.005 | 72,337  (1.82) | 0.789 | 0.054 | 38,254  (3.21) | 0.747 | 0.071 | 37,950 (3.22) | 0.748 | 0.072 |
|  | TRDW | 6,725  (2.51) | 0.723 | 0.064 | 1,062 (3.01) | 0.769 | 0.100 | 725  (3.72) | 0.734 | 0.112 | 395 (5.32) | 0.731 | 0.132 |
|  | VA | N/A | N/A | N/A | 23,960  (4.93) | 0.769 | 0.138 | 5,990  (8.11) | 0.728 | 0.168 | 1,446 (10.30) | 0.689 | 0.179 |
| Hospitalization with pneumonia requiring intensive services ore death  (COVER-I) | CUIMC | 27,356 (1.46) | 0.778 | 0.043 | 4,337 (3.25) | 0.777 | 0.081 | 3,354 (4.11) | 0.756 | 0.093 | 2,731 (4.907) | 0.734 | 0.100 |
|  | HIRA | 58,072 (0.85) | 0.858 | 0.035 | 48,057 (1.00) | 0.854 | 0.039 | 47,594 (1.01) | 0.856 | 0.040 | 1,985 (1.11) | 0.910 | 0.053 |
|  | SIDIAP | 415,119 (0.03) | 0.775 | 0.003 | N/A | N/A | N/A | N/A | N/A | N/A | N/A | N/A | N/A |
|  | TRDW | 6,725 (0.46) | 0.769 | 0.018 | 1,062 (0.47) | 0.816 | 0.083 | 725 (0.69) | 0.807 | 0.222 | 395 (1.27) | 0.779 | 0.230 |
|  | VA | N/A | N/A | N/A | 23,960  (1.22) | 0.805 | 0.043 | 5,990  (2.02) | 0.780 | 0.056 | 1,446 (2.63) | 0.763 | 0.058 |
| Fatality (COVER-F) | CUIMC | 27,356 (1.58) | 0.847 | 0.082 | 4,337 (7.89) | 0.843 | 0.320 | 3,354 (10.05) | 0.834 | 0.377 | 2,731 (12.27) | 0.82 | 0.400 |
|  | HIRA | 58,072 (2.28) | 0.851 | 0.099 | 48,057 (2.75) | 0.846 | 0.113 | 47,594 (2.78) | 0.846 | 0.114 | 1,985 (2.17) | 0.898 | 0.150 |
|  | SIDIAP | 415,119 (0.04) | 0.885 | 0.010 | 72,337 (0.60) | 0.919 | 0.068 | 38,254 | 0.895 | 0.082 | 37,950 (1.07) | 0.895 | 0.083 |
|  | TRDW | 6,725 (0.07) | 0.819 | 0.002 | 1,062 (0.09) | 0.970 | 0.015 | 725 (0.14) | 0.971 | 0.023 | 395 (0.25) | 0.959 | 0.030 |
|  | VA | N/A | N/A | N/A | 23,960 (0.72) | 0.809 | 0.036 | 5,990 (1.77) | 0.765 | 0.058 | 1,446 (2.97) | 0.717 | 0.068 |

# Appendix 1C: Receiver operating characteristic and calibration plots

The figures below show the receiver operating characteristic and calibration plots for patients with COVID-19 in 2020, all plots are available online: <http://evidence.ohdsi.org/Covid19CoverPrediction.>

| Model | Database | |
| --- | --- | --- |
| COVER-H | HIRA | |
|  | 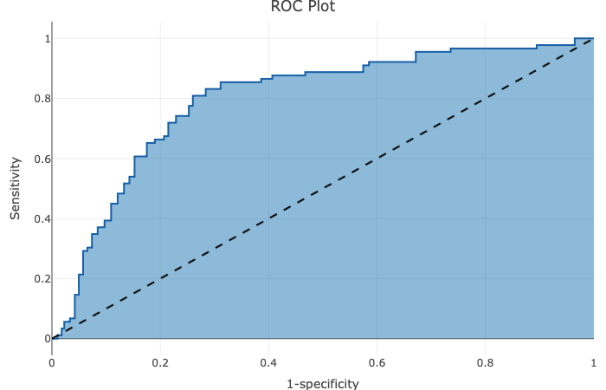 | 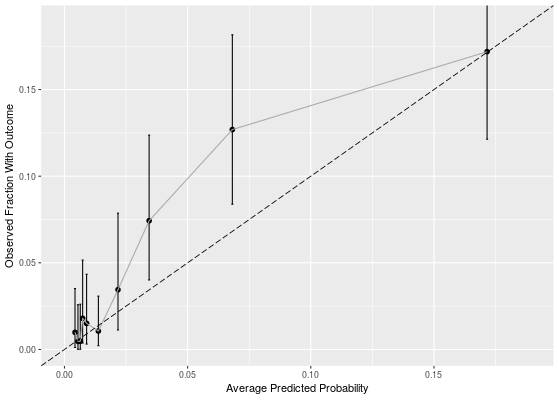 |
|  | SIDIAP | |
|  | 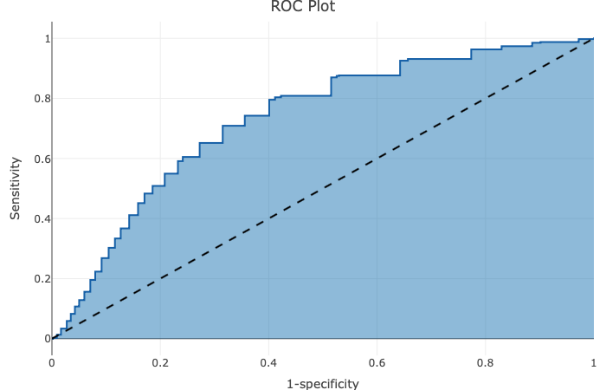 | 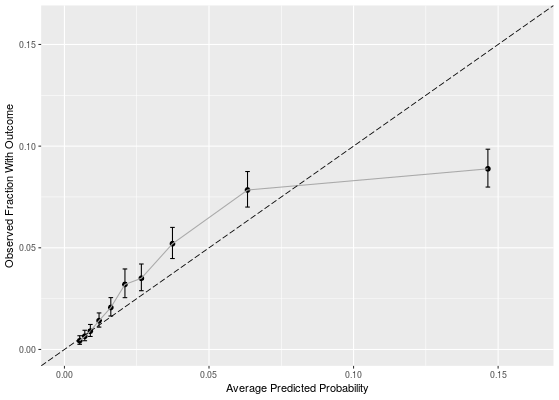 |
|  | TRDW | |
|  | 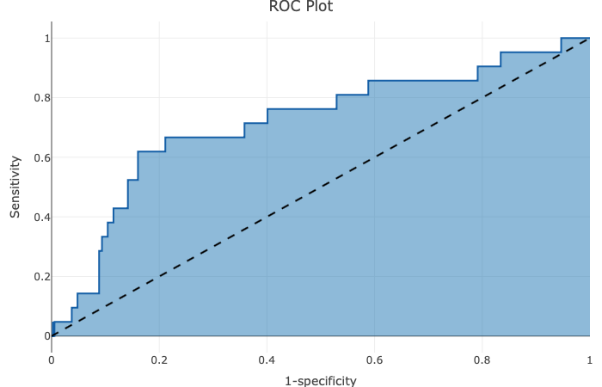 | 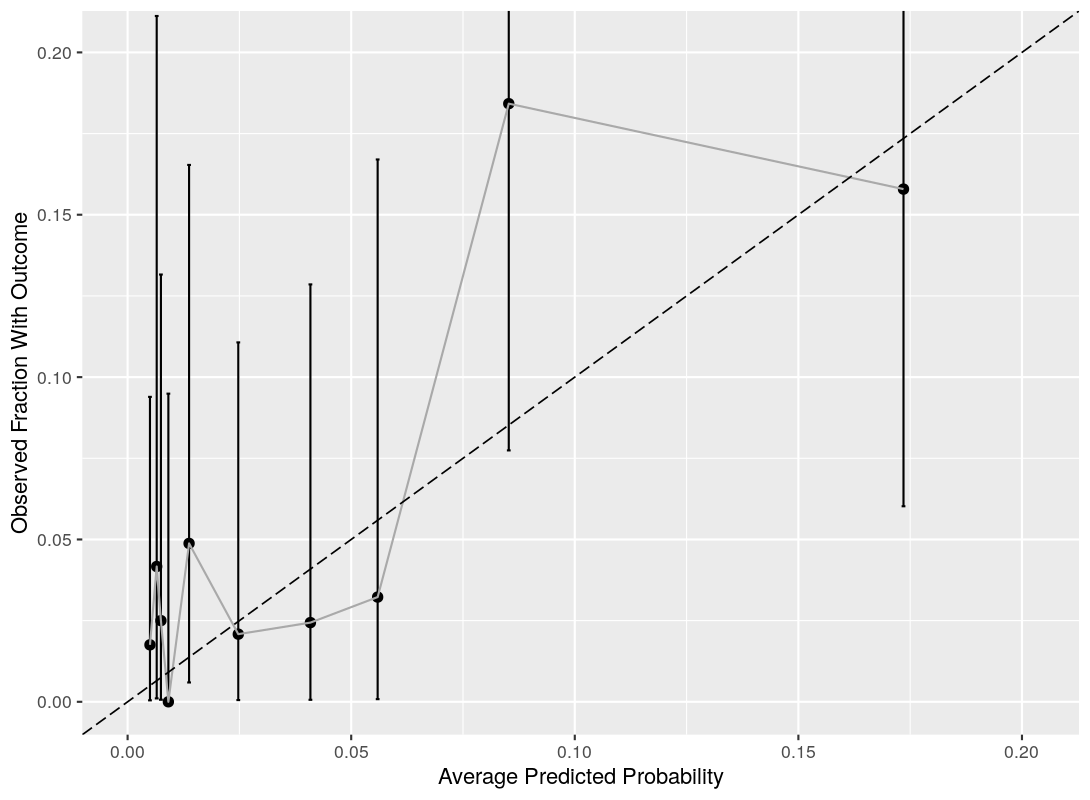 |
|  | VA | |
|  | 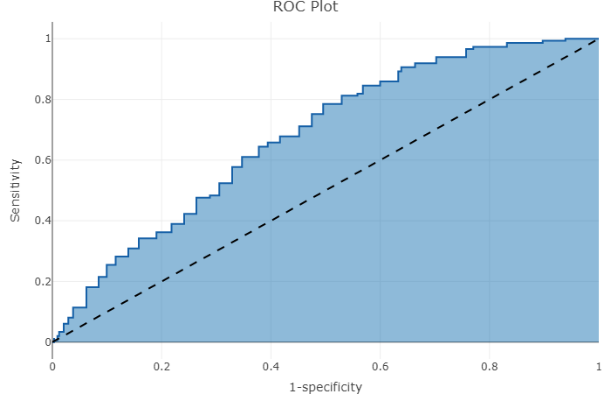 | 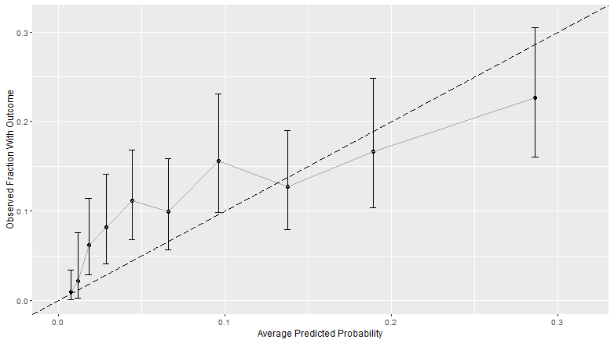 |
| COVER-I | CUIMC | |
|  | 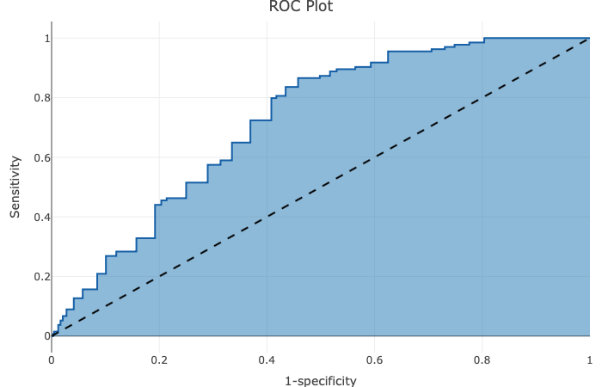 | 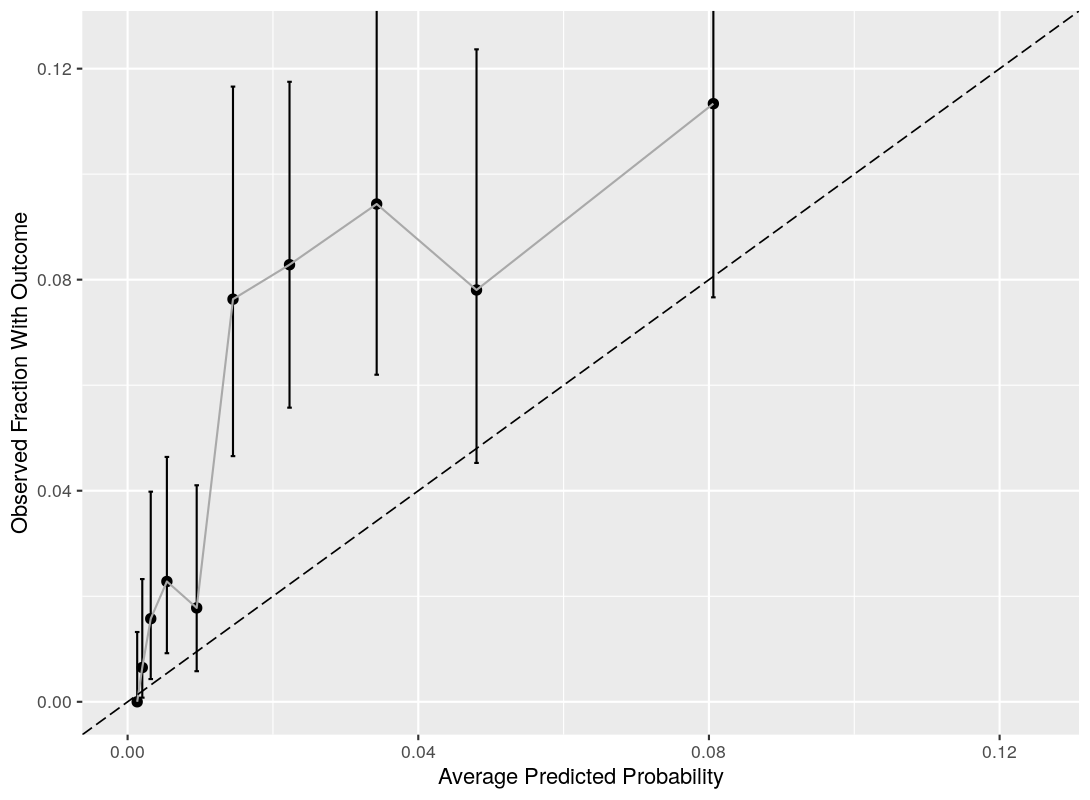 |
|  | HIRA |  |
|  | 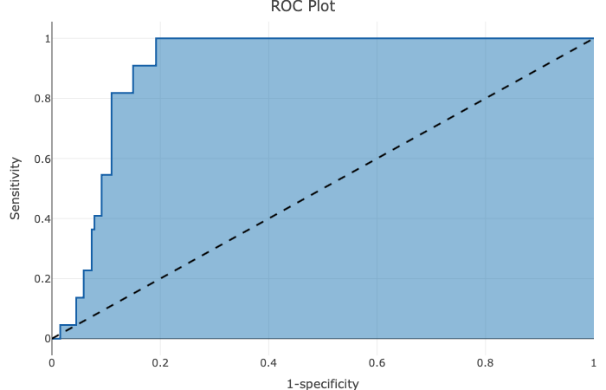 | 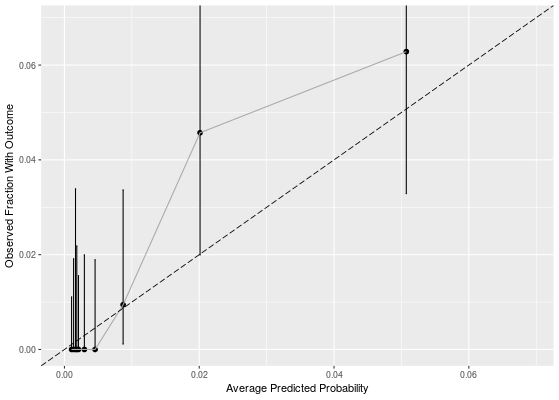 |
|  | VA | |
|  | 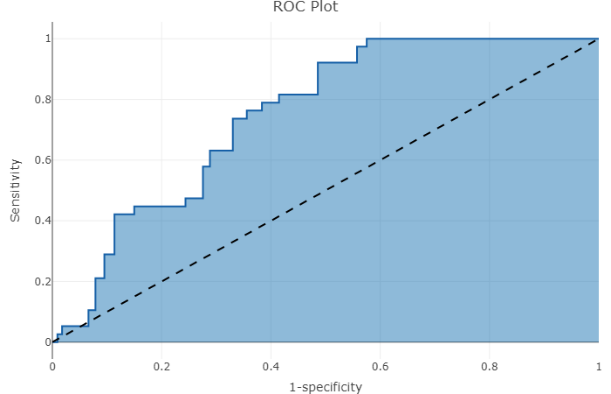 | 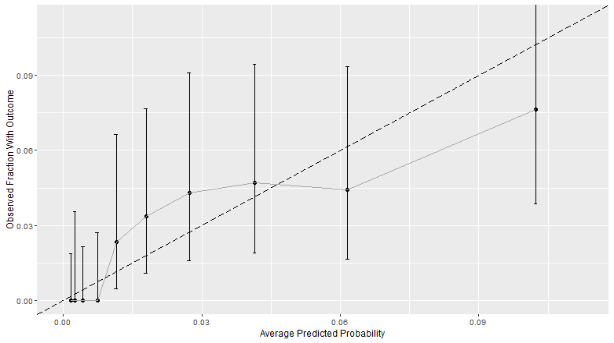 |
| COVER-F | CUIMC | |
|  | 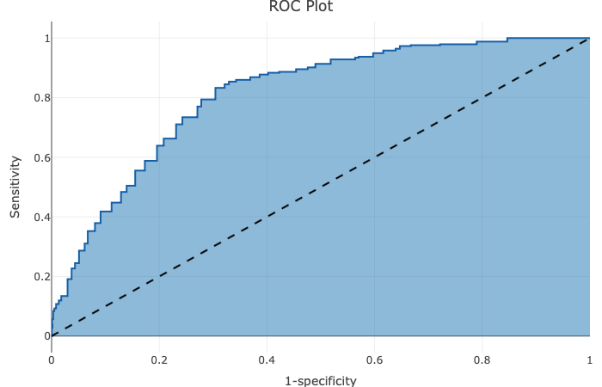 | 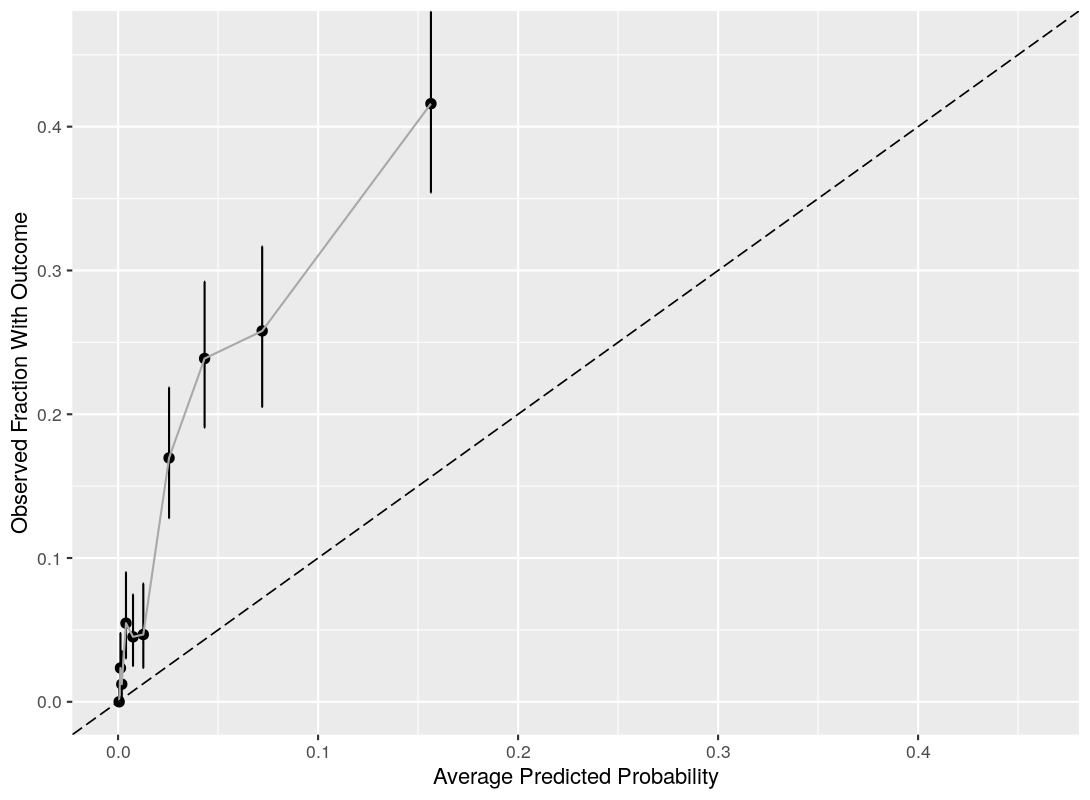 |
|  | HIRA | |
|  | 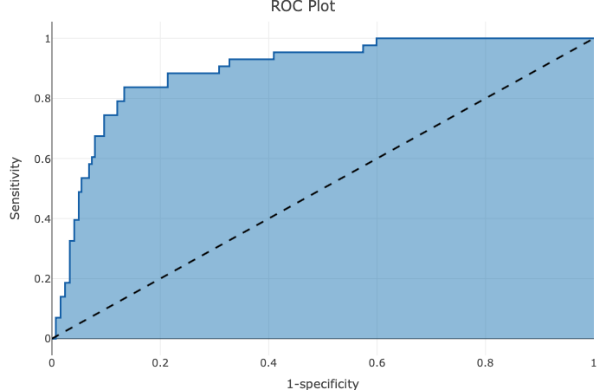 | 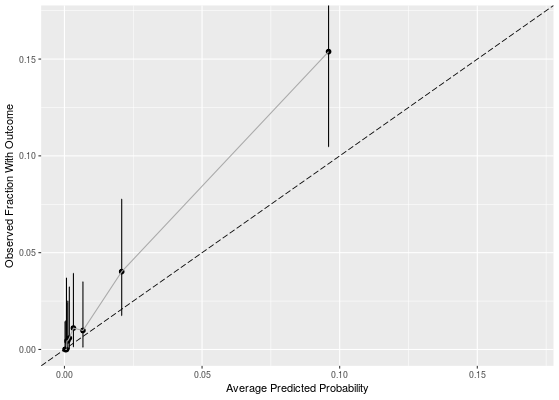 |
|  | SIDIAP | |
|  | 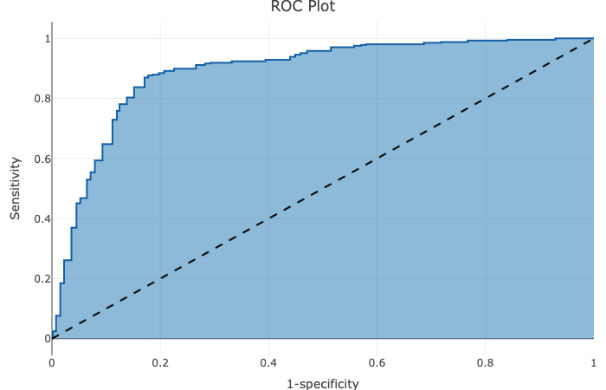 | 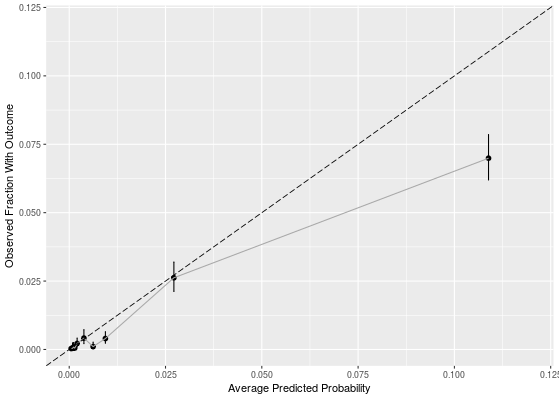 |
|  | VA | |
|  | 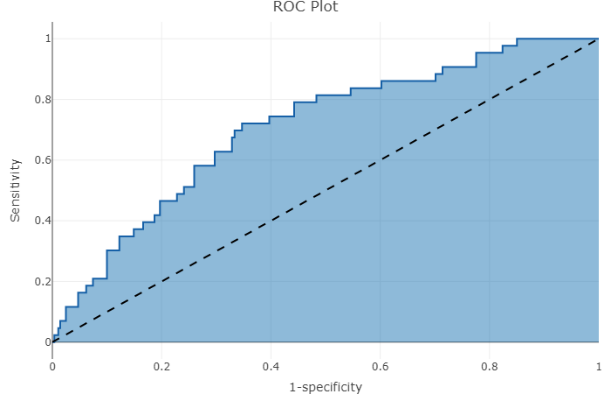 | 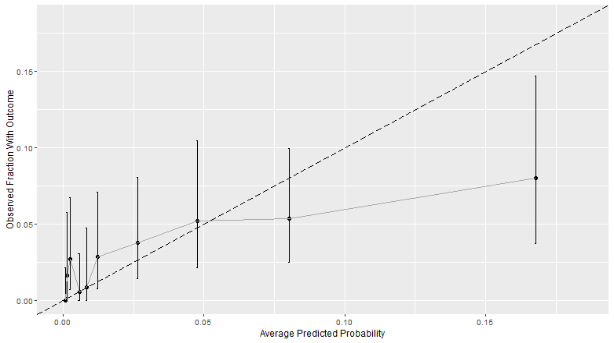 |
